# The Use of Glycomacropeptide in Patients with Phenylketonuria: A Systematic Review and Meta-Analysis

**DOI:** 10.3390/nu10111794

**Published:** 2018-11-18

**Authors:** Maria João Pena, Alex Pinto, Anne Daly, Anita MacDonald, Luís Azevedo, Júlio César Rocha, Nuno Borges

**Affiliations:** 1 Departamento de Biomedicina, Unidade de Bioquímica, Faculdade de Medicina, Universidade do Porto, 4200-319 Porto, Portugal; 2 Department of Dietetics, Birmingham Children’s Hospital, Birmingham B4 6NH, UK; alex.pinto@nhs.net (A.P.); a.daly3@nhs.net (A.D.); anita.macdonald@nhs.net (A.M.); 3 Faculty of Health & Human Sciences, University of Plymouth, Plymouth PL6 8BH, UK; 4 Faculdade de Medicina, Universidade do Porto, 4200-319 Porto, Portugal; lazevedo@med.up.pt; 5 Department of Community Medicine, Information and Health Decision Sciences (MEDCIDS), 4200-450 Porto, Portugal; 6 Center for Health Technology and Services Research (CINTESIS), 4200-450 Porto, Portugal; julio.rocha@chporto.min-saude.pt (J.C.R.); nunoborges@fcna.up.pt (N.B.); 7 Centro de Genética Médica Dr Jacinto de Magalhães, Centro Hospitalar Universitário do Porto, 4099-028 Porto, Portugal; 8 Centro de Referência na área das Doenças Hereditárias do Metabolismo, Centro Hospitalar Universitário do Porto—CHP EPE, 4099-001 Porto, Portugal; 9 Faculdade de Ciências da Nutrição e Alimentação, Universidade do Porto, 4200-465 Porto, Portugal

**Keywords:** phenylketonuria, glycomacropeptide, amino acids, phenylalanine, metabolic control, nutritional biomarkers, acceptability

## Abstract

In phenylketonuria (PKU), synthetic protein derived from L-amino acids (AAs) is essential in a low-phenylalanine (Phe) diet. Glycomacropeptide (GMP), an intact protein, is very low in Phe in its native form. It has been modified and adapted for PKU to provide an alternative protein source through supplementation with rate-limiting amino acids (GMP-AAs), although it still contains residual Phe. This review aims to systematically evaluate published intervention studies on the use of GMP-AAs in PKU by considering its impact on blood Phe control (primary aim) and changes in tyrosine control, nutritional biomarkers, and patient acceptability or palatability (secondary aims). Four electronic databases were searched for articles published from 2007 to June 2018. Of the 274 studies identified, only eight were included. Bias risk was assessed and a quality appraisal of the body of evidence was completed. A meta-analysis was performed with two studies with adequate comparable methodology which showed no differences between GMP-AAs and AAs for any of the interventions analysed. This work underlines the scarcity and nature of studies with GMP-AAs interventions. All were short-term with small sample sizes. There is a need for better-designed studies to provide the best evidence-based recommendations.

## 1. Introduction

Phenylketonuria (PKU, OMIM # 261200) is the most common inborn error of amino acid metabolism and is caused by a defect in phenylalanine (Phe) hydroxylase (PAH; EC 1.14.16.1) or in its cofactor, tetrahydrobiopterin (BH_4_). The resulting accumulation of Phe in blood and brain causes irreversible neurological impairment [1].

The low-Phe diet introduced in the 1950s by Dr. Horst Bickel was a milestone that allowed avoidance of severe complications for patients with PKU [2]. Untreated PKU may lead to irreversible intellectual disability, microcephaly, motor deficits, eczematous rash, autism, seizures, developmental problems, aberrant behaviour, and psychiatric symptoms [3]. Dietary treatment requires natural protein and Phe restrictions, together with synthetic protein substitutes that provide most of the nitrogen in the diet [4]. The availability of protein substitutes in Europe as well as the nutritional profile of protein substitutes available in Portugal were previously reported. Differences in the availability across European countries and nutritional inconsistencies were found [5].

Following Kure’s first description of the impact of BH_4_ in PKU in 1999 [6], pharmacological treatment with sapropterin has allowed a relaxation of dietary restrictions in a subgroup of patients, mainly those with mild or moderate PKU. This compound acts as a pharmaceutical chaperone [3], but almost all patients require a low-Phe diet supplemented with a synthetic protein derived from L-amino acids (AAs) [3]. PKU requires lifelong treatment in order to keep blood Phe control within acceptable target ranges, but dietary adherence is challenging, especially in adolescence and adulthood [7].

In the last decade, glycomacropeptide (GMP), a whey-based natural protein derived from the cheese manufacturing process, has been introduced for PKU [8]. It contains only residual amounts of Phe, tyrosine (Tyr), and tryptophan [9], and has many functional and physiological properties. It acts as a prebiotic, and has anti-inflammatory and nutraceutical properties, creating an attractive peptide for patients with inherited metabolic disorders as an alternative protein replacement for AAs [10]. GMP is an incomplete intact protein, but in PKU it is supplemented with any deficient amino acids (GMP-AAs) to offer a more nutritionally complete product [11].

In a preclinical study, wild-type and PKU mice were fed diets consisting of 20% protein from casein, AAs, or GMP-AAs. In this study, the GMP-AAs group showed similar growth and significantly reduced concentrations of Phe in plasma and brain compared to those fed by conventional sources [12]. Another study sought to evaluate the effect of three diets (GMP-AAs, AAs, and casein) on plasma amino acids, cytokines, fat and lean mass, and acute energy balance in PKU and wild-type mice. The PKU mice had growth and lean mass similar to the wild-type mice fed GMP-AAs or AAs. However, the GMP-AAs significantly reduced energy expenditure, food intake and plasma Phe concentrations in PKU mice, whereas AAs and casein induced metabolic stress [13]. Neurotransmitter concentrations and behavioural phenotype were found to be similar in PKU mice fed with either GMP-AAs or AAs [14]. In a further animal study, GMP-AAs showed prebiotic properties by positively modulating the gut microbiota, increasing short-chain fatty acids, and reducing inflammatory markers [15]. A study by Solverson et al. [16] reported potential long-term benefits for bone health using GMP-AAs. 

Overall, the studies in PKU mice showed a positive influence of GMP-AAs. However, scientific evidence from clinical studies that support the use of GMP-AAs as a major source of protein in PKU patients is less robust. Doubts still persist regarding the potential effect on patients of the residual Phe provided by GMP-AAs as well as how the nutritional biomarkers are influenced by GMP-AAs intake [10,17,18,19,20,21,22,23,24,25,26,27]. 

The primary aim was to systematically review the existing literature relating to the influence of residual Phe in GMP-AAs on blood Phe control. The secondary aims were to evaluate the impact on blood Tyr metabolic control, changes in nutritional biomarkers, and the acceptability or palatability of GMP products. 

## 2. Materials and Methods

### 2.1. Review Question

A systematic literature search was performed according to the Preferred Reporting Items for Systematic Reviews and Meta-Analysis (PRISMA) guidelines [28]. The protocol is registered with the “International prospective register of systematic reviews” (PROSPERO) with systematic review number CRD42018098873. 

Inclusion criteria included articles reporting observational or interventional studies. Articles of preclinical studies (defined as not providing clinical outcome data) or abstracts were excluded. Based on the Patients, Intervention, Comparator, Outcomes (PICO) approach, the patients/populations under study included male and female subjects diagnosed with PKU, with ages ranging from infancy to adulthood, under treatment with diet only or diet plus sapropterin, and who were willing to take GMP-AAs or AAs as their primary nitrogen source or cheese made of GMP. Exclusion criteria for patients/populations were pregnancy and no dietary treatment.

### 2.2. Search Strategy

Eligible literature published from 2007 to June 2018 was obtained from PubMed, CENTRAL Cochrane Library, Scopus and Web of Science. Studies were sought with the following terms: PubMed query—(“Phenylketonuria” [All fields] OR “Phenylketonuria” [MeSH TERM] OR “PKU” [All fields]) AND (“Glycomacropeptide” [All fields] OR “kappa-casein glycomacropeptide” [Supplementary Concept] OR “caseinomacropeptide” [Supplementary Concept] OR GMP [All fields]); CENTRAL Cochrane Library query—#1. “phenylketonuria”: ti,ab,kw, #2. MeSH descriptor: [Phenylketonurias] explode all trees, #3. “PKU”, #4. #1 or #2 or #3, #5. “glycomacropeptide”, #6. #4 and #5; Scopus query—(“Phenylketonuria” OR “PKU”) AND “Glycomacropeptide”; Web of Science query—#1. TS = Phenylketonuria, #2. TS = PKU, #3. TS = Glycomacropeptide, #4. #1 OR #2, #5. #3 AND #4.

### 2.3. Study Selection

The first stage in the process was to review the titles and abstracts of the studies. These were screened independently by two investigators (M.J.P. and A.P.) based on the inclusion and exclusion criteria. Articles of overlapping participants were also screened and considered independent of the “parent” study. A record number was assigned to each included study. Any disagreements were overcome by consensus. When a research study was considered eligible, it was selected for full text review. Of the 274 studies identified, eight were eligible for inclusion.

### 2.4. Data Extraction

Data was extracted by two independent investigators (M.J.P. and A.P.): author and year, country, study design, length of intervention, sample size, patients’ characteristics, intervention features, comparator features, and outcomes (blood Phe levels, blood Tyr levels, blood urea nitrogen (BUN), glucose levels and acceptability/palatability). For all included studies, mean ± standard deviation (SD) or standard error of mean (SEM) or median and interquartile range (IQR) were used for data extracted.

### 2.5. Quality Appraisal

The quality of all included studies was assessed using the Grading of Recommendations Assessment, Development and Evaluation (GRADE) system [29]. The GRADE ranks as follows: not serious, serious and very serious. The GRADE level of evidence was determined independently by two authors (M.J.P. and L.A.), and consensus was achieved by discussion.

### 2.6. Assessment of Risk of Bias

The Cochrane Collaboration’s domain-based evaluation tool as described in Chapter 8, Section 8.5, in the Cochrane Handbook for Systematic Reviews of Interventions was used to assess risk of bias of randomised clinical trials (RCTs) [30]. This tool comprises six domains: random sequence generation (selection bias); allocation concealment (selection bias); blinding of participants and personnel (performance bias); blinding of outcome assessment (detection bias); incomplete outcome data (attrition bias), and selective reporting (reporting bias). Each RCT was rated as low risk, unclear risk or high risk of bias. 

The Risk of Bias in Non-Randomised Studies of Interventions (ROBINS-I) assessment tool was used for non-randomised studies (observational studies). This tool includes seven specific bias domains, pre-intervention and post-intervention [31]. The domains are: (1) confounding; (2) selection of participants; (3) classification of intervention; (4) deviation from interventions; (5) missing outcome data; (6) measurement of outcomes; and (7) selection of reported result overall. Risk of bias was rated as 0—no information; 1—low risk; 2—moderate risk; 3—serious risk; and 4—critical risk.

Two authors independently assessed risk of bias (M.J.P. and L.A.) of the included articles. Disagreements were managed by consensus.

### 2.7. Data Analysis

Meta-analysis was performed using Review Manager Version 5.3 (The Nordic Cochrane Centre, The Cochrane Collaboration 2014, Portland, OR, USA).

Our primary question was about the effect of GMP intervention on altering blood Phe concentrations in PKU. Due to absence of statistical information and assuming that randomisation was well conducted, we compared the final values of blood Phe. The same approach was applied to the secondary outcomes (blood Tyr, BUN, glucose). The secondary outcome acceptability/palatability was compiled in a table.

In two RCTs with sufficient methodological similarity [18,22], a meta-analysis was carried out. The study of Ahring et al. [18] tested four drink mixtures (DMs 1–4), consisting of GMP or AAs or a combination. For the purposes of analysis, we only considered DM3 and DM4. In the same study, the values of BUN and glucose were available in mmol/L which were converted to mg/dL. In these two studies, GMP-AAs provided 1.8 mg Phe/g of protein equivalent. A forest plot was generated and calculated the mean difference (MD) as the effect measure. We combined the MD with the use of the random-effects model. The degree of statistical heterogeneity between studies was assessed with the use of the *I*^2^ statistic. We reported statistical heterogeneity as important if the *I*^2^ statistic was ≥40%, according to the Cochrane guidelines. Significance was set at the level of *P*-value less than 0.05.

## 3. Results

### 3.1. Study Selection

**Figure 1:**
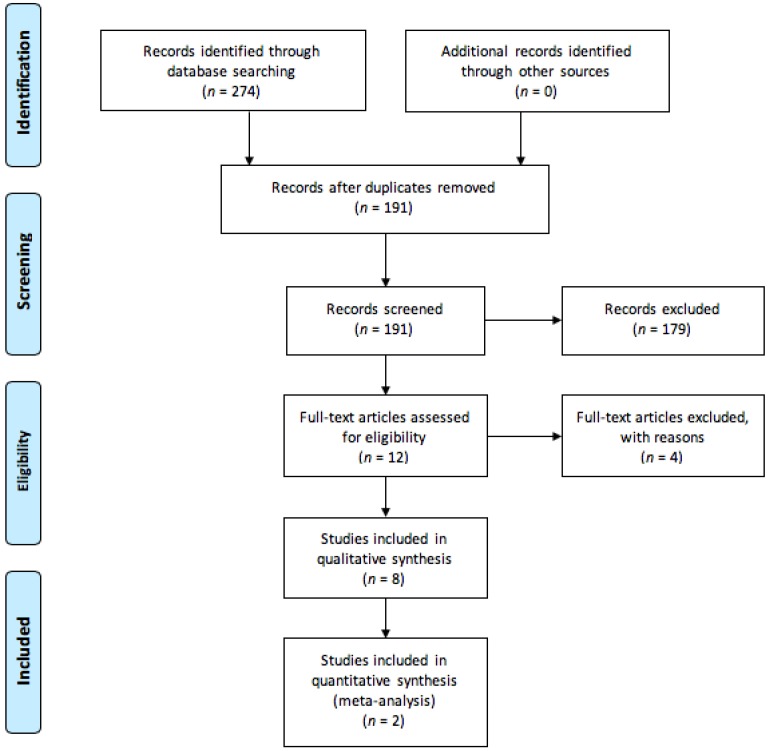
Preferred Reporting Items for Systematic Reviews and Meta-Analysis (PRISMA) study flow diagram describing process of study selection. Reviews or preclinical studies (defined as not providing clinical outcome data) and abstracts were excluded. Full-text articles that provided no outcome of interest were also excluded.

Figure 1 describes the process of study selection according to PRISMA. The first literature search identified 274 articles. Initial screening identified 12 papers for full text review. From this, 4 were eliminated as they failed to meet the exclusion criteria. Eight studies were eligible for the systematic review and meta-analysis was performed for only two studies.

### 3.2. Study Characteristics

Table 1 summarizes the main characteristics of all included articles. Only two studies were considered RCTs with crossover according to the Consolidated Standards of Reporting Trials (CONSORT) guidelines [32] and the remaining six studies were as follows: two crossover clinical studies, two clinical studies, one retrospective study, and one cross-sectional study. Four studies were conducted in the United States at the University of Wisconsin–Madison. The remaining four studies were performed in the United Kingdom, Portugal, Denmark, and Egypt. Studies were published between 2007 and 2018, with the vast majority published since 2010.

The total sample size of the included articles was 139 participants, since the participants of the study of MacLeod et al. [20] were recruited from the “parent” study of van Calcar et al. [17]. The largest trial conducted in patients with PKU taking GMP-AAs was the study by Ney et al. [22], with a total of 30 participants. The participants in most of the studies were adults, with the exception of the articles from Zaki et al. [21] and Daly et al. [23], that recruited children with PKU. Regarding the PKU phenotype, the predominant form was classical PKU. 

### 3.3. Treatment and Outcome Measures

Table 2 illustrates the characteristics of treatment and outcome measures. The length of intervention is quite variable across studies, ranging from eight days to twenty months. In the studies of van Calcar et al. [17] and MacLeod et al. [20], subjects consumed the AAs diet or the GMP-AAs diet for four days. In the work performed by Zaki et al. [21], the study was divided into two periods, each lasting nine weeks each. In one of the periods, children were given 50% of their total protein substitute as GMP made with cheese and the remaining 50% was given in the form of AAs. In the other period, the total protein substitute was taken in the form of AAs. In the study of Ney et al. [22], subjects consumed for three weeks each, in a random order, AAs or GMP-AAs, separated by a washout period of three weeks with AAs. In the work of Daly et al. [23], 12 children received GMP-AAs (partially or fully to replace AAs but individually titrated according to their blood Phe control) and 9 subjects received AAs as their protein substitute. In the retrospective study conducted by Pinto et al. [24] with 11 subjects, GMP-AAs partially or fully substituted AAs. In the study performed by Ahring et al. [18], subjects tested four DMs (1–4) in a random order at each visit (DM1 = GMP; DM2 = AAs (equivalent amino acid profile to DM1); DM3 = GMP + AAs; DM4 = AAs (equivalent amino acid profile to DM3 but without Phe). 

Considering the outcome measures, only the study by Lim et al. [10] evaluated acceptability. The remaining studies used blood Phe as a primary outcome measure. Blood Tyr was measured in all studies, with exception of the studies of Lim et al. [10] and Zaki et al. [21]. BUN was measured in the studies of van Calcar et al. [17], Ney et al. [22], and Ahring et al. [18]. In the studies of Zaki et al. [21] and Pinto et al. [24], only the values of urea were available. Glucose was measured in the studies of van Calcar et al. [17], Ney et al. [22], Pinto et al. [24], and Ahring et al. [18]. Acceptability was assessed by all apart from Pinto et al. [24].

### 3.4. Acceptability/Palatability of GMP Products

Acceptability is a hedonic response affected by the organoleptic properties of products, among others. Measuring acceptability is both subjective and complex, with many different methodologies available. Acceptability of GMP products was determined based on different methodologies as illustrated in Table 3. The included studies showed that GMP products were well accepted by patients. There is evidence suggesting that GMP products based on natural protein source are more palatable than protein substitutes based on mono amino acids. However, it is important to highlight the lack of uniformity in the methods used to evaluate this parameter. The presentation form of the products was also variable, some studies used solid food whereas other studies used only drinks. 

### 3.5. Quality Appraisal

Using the GRADE system, inconsistency and imprecision were the most common reasons for downgrading (Table 4).

### 3.6. Assessment of Risk of Bias

**Figure 2:**
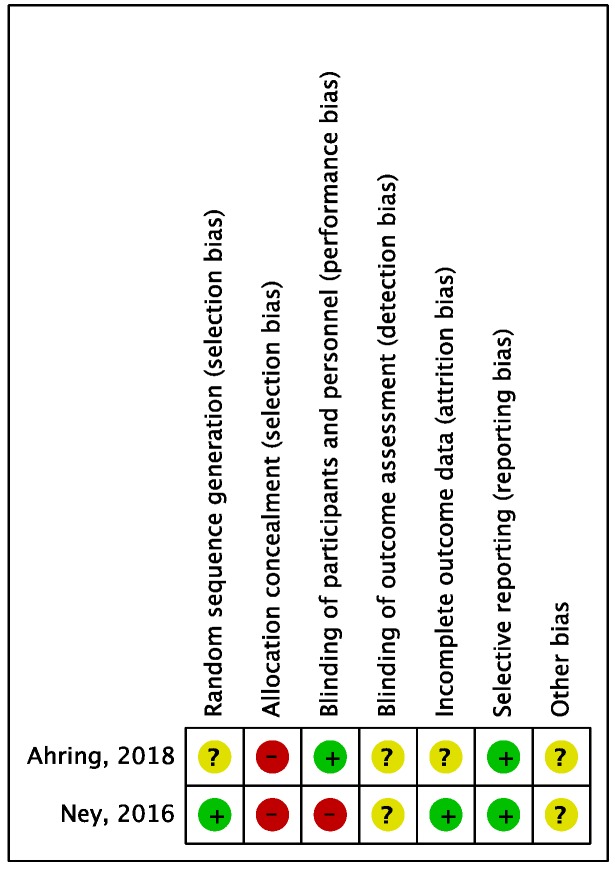
Risk of bias summary across randomised controlled trials. Low risk of bias: green “+”; Unclear risk of bias: yellow “?”; High risk of bias: red “−”.

**Figure 3:**
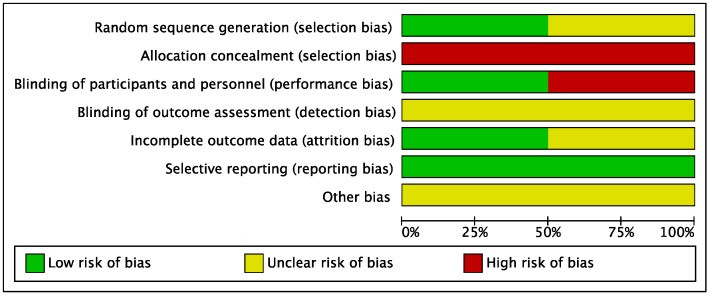
Risk of bias graph across randomised controlled trials. Low risk of bias: green; Unclear risk of bias: yellow; High risk of bias: red.

Risk of bias for RCTs was evaluated according to the Cochrane guidelines (Figure 2 and Figure 3). Only two out of eight studies were considered RCTs. For the domain random sequence generation (selection of bias), 1/2 rated as unclear and 1/2 rated as low; for the domain allocation concealment (selection bias), 2/2 rated as high; for the domain blinding of participants and personnel (performance bias), 1/2 rated as low and 1/2 rated as high; for the domain blinding of outcome assessment (detection bias), 2/2 rated as unclear; for the domain incomplete outcome data (attrition bias), 1/2 rated as unclear and 1/2 rated as low; for the domain selective reporting (reporting bias), 2/2 rated as low.

Risk of bias for observational studies was evaluated using ROBINS-I tool (Table 5). In domains 1 and 2, 5/5 were rated as serious; in domains 3, 4 and 5, 5/5 rated as low; in domains 6 and 7, 4/5 were rated as low and 1/5 rated as serious; and overall, 5 of 5 were rated as moderate risk of bias. All studies provided sound evidence for non-randomised studies but cannot be considered comparable to well-performed randomised trials.

### 3.7. Meta-Analysis

**Figure 4:**
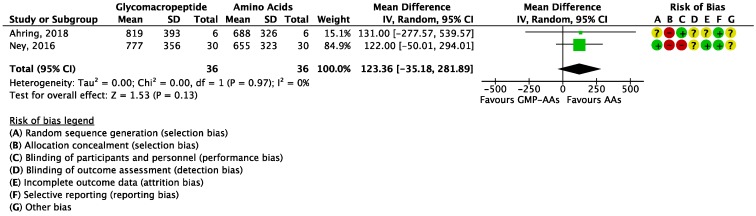
Forest plot of studies with data on the effect of glycomacropeptide interventions on blood phenylalanine levels. The analysis included data from two studies with a total of 72 participants. AAs: synthetic protein derived from L-amino acids; CI: confidence interval; df: degrees of freedom; GMP-AAs: glycomacropeptide supplemented with amino acids; IV: intravitreal; SD: standard deviation.

Focusing on the primary outcome (blood Phe levels), the meta-analysis showed no significant differences between GMP-AAs and AAs (MD = 123.36 μmol/L (−35.18, 281.89); *I*^2^ = 0%; *P* = 0.13; two studies; *N* = 72 participants; Figure 4), although a tendency to lower Phe concentrations in patients treated with AAs was observed.

**Figure 5:**
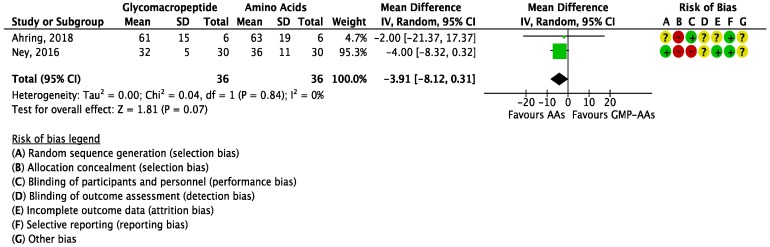
Forest plot of studies with data on glycomacropeptide interventions on blood tyrosine levels. The analysis included data from two studies with a total of 72 participants.AAs: synthetic protein derived from L-amino acids; CI: confidence interval; df: degrees of freedom; GMP-AAs: glycomacropeptide supplemented with amino acids; IV: intravitreal; SD: standard deviation.

The overall treatment effect on blood Tyr levels was not statistically significant (MD = −3.91 μmol/L (−8.12, 0.31); *I*^2^ = 0%; *P* = 0.07; two studies; *N* = 72 participants; Figure 5) and patients treated with AAs tended to have higher levels of Tyr.

**Figure 6:**
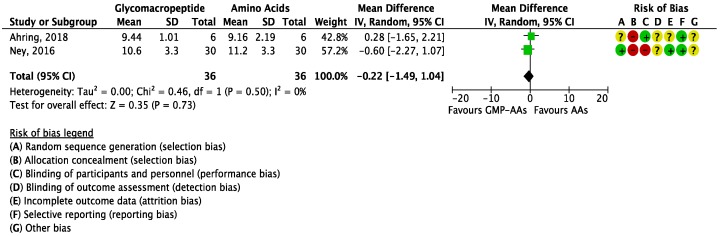
Forest plot of studies with data on the effect of glycomacropeptide interventions on blood urea nitrogen. The analysis included data from two studies with a total of 72 participants.AAs: synthetic protein derived from L-amino acids; CI: confidence interval; df: degrees of freedom; GMP-AAs: glycomacropeptide supplemented with amino acids; IV: intravitreal; SD: standard deviation.

**Figure 7:**
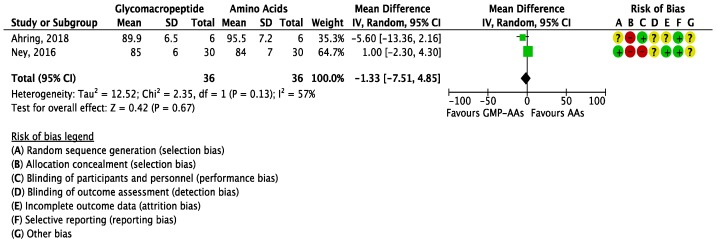
Forest plot of studies with data on the effect of glycomacropeptide interventions on glucose levels. The analysis included data from two studies with a total of 72 participants. AAs: synthetic protein derived from L-amino acids; CI: confidence interval; df: degrees of freedom; GMP-AAs: glycomacropeptide supplemented with amino acids; IV: intravitreal; SD: standard deviation.

The meta-analysis for BUN reported no significant differences between GMP-AAs and AAs (MD = −0.22 mg/dL (−1.49, 1.04); *I*^2^ = 0%; *P* = 0.73; two studies; *N* = 72 participants; Figure 6); nor did the meta-analysis for glucose levels (MD = −1.33 mg/dL (−7.51, 4.85); *I*^2^ = 57%; *P* = 0.67; two studies; *N* = 72 participants; Figure 7). 

When analysing BUN, the value of SD of DM4 was imputed since no value was reported. It was calculated from the arithmetic mean of SD of DM2 from baseline and final and DM4 from baseline. 

The studies included in this meta-analysis were quite consistent in all outcomes as a result of *I*^2^ values, a measure of heterogeneity. Nevertheless, the length of study was different between studies, and in the study of Ahring et al. [18], patients had high blood Phe levels at the start of the study and this aspect could have masked the results.

## 4. Discussion

This is the first systematic review and meta-analysis addressing the use of GMP in the nutritional management of PKU. This study was designed with the aim of reviewing the current literature on the use of GMP in PKU and the effect of residual Phe in GMP on blood Phe control, biochemical status, and palatability. 

Overall, pooled results based on two RCTs reported no significant effect for all outcome measures. For blood Phe control, in the adult studies, meta-analysis showed a tendency in favour of AAs despite no clinical significance. AAs have no added Phe and the effect of the extra Phe provided by the GMP-AAs may have been masked as adult subjects started with higher baseline blood Phe [18]. Children maintain lower blood Phe target concentrations so may have less tolerance with additional Phe sources. In addition, fever and recurrent infections are more likely to impact on blood Phe control in children [33]. It is well known that administration of AAs during any acute phases suppresses Phe levels, improving metabolic control [3]. So far, it remains undocumented if GMP-AAs intake can suppress the rise in Phe levels in a similar way to AAs, as little is known about the kinetics of GMP-AAs in PKU. Additionally, the impact of GMP-AAs on glucose metabolism and anabolic pathways remains to be studied, and ultimately an influence on Phe levels cannot be dismissed. The studies by Zaki et al. [21] and Daly et al. [23], investigated the effect on blood Phe control using two different formulations of GMP in 10 and 22 children with PKU, respectively. The different interventions in these two studies prevented subgroup analysis, which would have enabled a better understanding of the impact of GMP in the paediatric population versus adulthood. So far, the research about the effects of GMP in children is still insufficient to advocate its use as a safe alternative to the traditional treatment. A systematic review with three trials evaluating the use of protein substitutes in PKU concluded that the current evidence is scarce and until robust evidence from RCTs is obtained, the use of all protein substitutes should be monitored carefully [34]. Nevertheless, the clinical use of AAs for several decades counterweighs the scarcity of scientific evidence emerging from RCTs [3].

When we performed meta-analysis on the effect of GMP-AAs versus AAs on blood Tyr levels, patients treated with AAs tended to have higher levels of Tyr. Tyr is considered a conditionally essential amino acid in PKU since it is produced from Phe and without treatment with a Tyr-supplemented protein substitute, Tyr deficiency is seen. The study of Ney et al. [22] reported that despite significantly higher intakes of Tyr in patients consuming a low-Phe diet in combination with AAs when compared to GMP-AAs, fasting plasma levels of Tyr were not statistically different. Moreover, the study of Pinto et al. [24] showed an increase in blood Tyr (even when dietary Tyr intake was lower) when patients consumed GMP-AAs.

A study performed in PKU mice showed that GMP-AAs acted as a prebiotic [15], shaping the gut microbiota. The GMP-AAs effects on gut microbiota may influence Tyr bioavailability [26]. Tyr is one of the amino acids with the lowest solubility [35], which can interfere with gut absorption [26].

For BUN and glucose levels, no conclusions can be reached. Subjects in the studies had similar protein intakes, irrespective of taking GMP-AAs or AAs. BUN is an indicator of the relationship between nutritional status and protein metabolism of patients [36]. In the study by van Calcar et al. [17], performed with 11 subjects, BUN was significantly lower and plasma insulin was higher when measured 2.5 h after eating a breakfast containing GMP-AAs. Glycaemia is known to be influenced by amino acid intake [37]. 

A further objective was to evaluate the acceptability/palatability of GMP products. Despite the different approaches used to measure acceptability in the included studies [10,17,18,20,21,22,23], GMP products were well accepted by patients. A very recent study from Proserpio et al. [38] published after our literature selection sought to explore the liking of low-Phe products (GMP products versus AAs) as well as to obtain a sensory description of them using the check-all-that-apply (CATA) method in 86 subjects with PKU in an ambulatory setting. The CATA questionnaire is a rapid sensory profiling approach to characterize foods based on sensory attributes. This is the first evidence of the sensory properties of GMP products in PKU subjects. The study included eight samples: four GMP products and four AAs flavoured with neutral, chocolate, strawberry, and tomato aromas. GMP products flavoured with chocolate and strawberry aromas were the most appreciated. The CATA method appears as a suitable method to fine-tune organoleptic properties to help improve dietary adherence. Nevertheless, this study does not provide data about the long-term acceptance of GMP products with patients and whilst the palatability of protein substitutes is important it is essential to assess the impact on metabolic control of any formulation that provides a source of Phe in all age groups and categories of patients with PKU. 

This systematic review has several strengths and limitations. The main strength is that it provides a compilation of the available evidence of GMP interventions and gives an overview of the current status. This will help in PKU guideline production. This study unveils the main flaws in the design of GMP interventions. First, among the eight studies included in this work, only two were RCTs, studies at the top of the hierarchy of evidence. Although high-quality observational studies can also produce comparable responses, well-conducted RCTs are still the gold-standard of evidence [39]. Secondly, all studies consisted of small sample sizes but in the context of PKU this cannot be undervalued due to the rarity of the disorder. Moreover, the RCT studies were short-term, and the adults did not have good metabolic control at baseline and had variable phenotypic presentations. 

The present systematic review and meta-analysis raises important aspects in the scope of PKU research. It would be ideal to create a group with PKU experts to develop standards in planning and designing better-quality studies for protein substitute research. However, it should be acknowledged that RCTs on protein substitutes are difficult to conduct due to food neophobia and poor acceptability of protein substitutes. 

## 5. Conclusions

The two studies that qualified for comparable investigation failed to show any reduction in plasma Phe, despite GMP-AAs providing 1.8 mg Phe/g of protein equivalent. This might be explained by the small number of available studies, small sample sizes, and short lengths of study. Considering that PKU is a chronic disease and requires lifelong treatment, further long-term research is warranted to understand in depth the safety and health benefits of GMP in the context of PKU. In the interim, the use of GMP in children should be carefully managed.

## Figures and Tables

**Table 1:** Characteristics of studies included in the systematic review.

Author, Year (Ref.)	Country	Study Design	Sample Size (*N*)	Age (Range)—Years	Gender	PKU Phenotype
Lim et al. 2007 [10]	United States	Cross-sectional study	49	12–42	N/A	N/A
van Calcar et al. 2009 [17]	United States	Crossover clinical study	11	23 ± 7 (11–31)	4 F; 7 M	10 classical; 1 variant form
MacLeod et al. 2010 [20]	United States	Crossover clinical study	11	23 ± 7 (11–31)	4 F; 7 M	11 classical
Zaki et al. 2016 [21]	Egypt	Clinical study	10	6.73 [5.02; 11.79]	4 F; 6 M	10 classical
Ney et al. 2016 [22]	United States	Randomised crossover clinical trial	30	15–49	18 F; 12 M	20 classical; 10 variant form
Daly et al. 2017 [23]	United Kingdom	Clinical study	22	11 (6–16)	9 F; 13 M	N/A
Pinto et al. 2017 [24]	Portugal	Retrospective, longitudinal study	11	27 ± 10 (13–42)	8 F; 3 M	6 classical; 4 mild; 1 HPA
Ahring et al. 2018 [18]	Denmark	Randomised crossover clinical trial	8 ^1^	33.25 ± 11.21 (15–48)	7 F; 1 M	8 classical

F: female; HPA: hyperphenylalaninemia; M: male; N/A: not available; PKU: phenylketonuria. Data are presented as mean ± standard deviation or median [interquartile range]. ^1^ Initial sample size was of eight patients but only six patients completed the study.

**Table 2:** Characteristics of treatment and outcome measures of the included studies.

Author, Year (Ref.)	Length of Intervention	Intervention	Comparator	Primary Outcome	Secondary Outcomes
Lim et al. 2007 [10]	N/A	GMP-AAs	AAs	N/A	Acceptability *
van Calcar et al. 2009 [17]	Two treatments for four days each: AAs (days 1–4) and GMP-AAs (days 5–8)	Period I—0% GMP-AAs; Period II—100% GMP-AAs; 11 patients	Period I—100% AAs; Period II—0% AAs; 11 patients	Blood Phe	Blood Tyr BUN Glucose Acceptability *
MacLeod et al. 2010 [20]	Two treatments for four days each: AAs (days 1–4) and GMP-AAs (days 5–8)	Period I—0% GMP-AAs; Period II—100% GMP-AAs; 11 patients	Period I—100% AAs; Period II—0% AAs; 11 patients	Blood Phe	Blood Tyr Acceptability *
Zaki et al. 2016 [21]	Eighteen weeks	Period I—50% GMP; Period II—0% GMP; 10 patients	Period I—50% AAs; Period II—100% AAs; 10 patients	Blood Phe	Urea/BUN Acceptability *
Ney et al. 2016 [22]	Eleven weeks	Three weeks each of GMP-AAs or AAs; 15 patients in each arm	Three weeks each of GMP-AAs or AAs; 15 patients in each arm	Blood Phe	Blood Tyr BUN Glucose Acceptability *
Daly et al. 2017 [23]	Twenty-six weeks	12 patients—GMP-AAs	9 patients—AAs	Blood Phe	Blood Tyr Acceptability *
Pinto et al. 2017 [24]	Twenty months	11 patients—GMP-AAs	11 patients—AAs	Blood Phe	Blood Tyr Urea/BUN Glucose
Ahring et al. 2018 [18]	Four visits, analysis at five timepoints (0, 15, 30, 60, 120 and 240 min)	6 patients tested the four DMs (DM1 = GMP; DM3 = GMP + AAs)	6 patients tested the four DMs [DM2 = AAs (equivalent amino acid profile to DM1); DM4 = AAs (equivalent amino acid profile to DM3 but without Phe)]	Blood Phe	Blood Tyr BUN Glucose Acceptability *

AAs: synthetic protein derived from L-amino acids; BUN: blood urea nitrogen; DM: drink mixture; GMP: glycomacropeptide; GMP-AA: glycomacropeptide supplemented with amino acids; N/A: not available; Phe: phenylalanine; PKU: phenylketonuria; Tyr: tyrosine. * The results of acceptability are shown in Table 3.

**Table 3:** Acceptability of GMP products versus AAs.

Author, Year (Ref.)	Method	Number of Items Evaluated	Type of Items Evaluated	Main Findings
Lim et al. 2007 [10]	Five-point hedonic scale ^1^	Seven products (five GMP-AAs and two AAs)	GMP-AAs (strawberry pudding, strawberry fruit leather, chocolate beverage, snack crackers, orange sports beverage) and AAs (crackers, chocolate beverage)	Decreasing order of overall acceptability—strawberry pudding (4.2 ± 0.9), snack cracker (3.6 ± 1.4), strawberry fruit leather (3.4 ± 1.0), chocolate beverage (3.3 ± 1.0), orange sports beverage (3.3 ± 1.1), AAs in crackers (2.9 ± 1.3), AAs in a chocolate beverage (2.5 ± 1.4)
van Calcar et al. 2009 [17]	No methodology described	Six GMP-AAs and subject’s usual AAs	GMP-AAs (orange-flavoured sports beverage, chocolate-flavoured or caramel-flavoured beverage, chocolate or strawberry pudding, cinnamon crunch bar) and subject’s usual AAs	After consuming the GMP-AAs diet for four days, 10 of 11 subjects claimed that the GMP-AAs products were superior in sensory qualities to their usual AAs. Moreover, at the end of the study, 6 of 7 adults expressed a strong preference to consume GMP-AAs products rather than their usual AAs
MacLeod et al. 2010 [20]	Four questions, motivation-to-eat VAS questionnaires	Six GMP-AAs and subject’s usual AAs	GMP-AAs (orange-flavoured sports beverage, chocolate-flavoured or caramel-flavoured beverage, chocolate or strawberry pudding, cinnamon crunch bar) and subject’s usual AAs	The motivation-to-eat VAS profiles were not significantly different at any timepoint between the AAs (day 4) and GMP-AAs (day 8)
Zaki et al. 2016 [21]	Questionnaire	N/A	N/A	Throughout the study, all patients preferred the diet supplemented with GMP over the classical AAs due to better taste and satiety
Ney et al., 2016 [22]	Six-question survey and six-point scale ^2^	Fifteen AAs and N/A the exact number of GMP-AAs	N/A	AAs vs GMP-AAs (1) 3.97 ± 0.24 vs 4.90 ± 0.18, *P* = 0.001 (2) 4.79 ± 0.22 vs 5.07 ± 0.16, *P* = 0.366 (3) 4.50 ± 0.25 vs 4.86 ± 0.19, *P* = 0.172 (4) 4.19 ± 0.18 vs 4.69 ± 0.16, *P* = 0.019 (5) 3.83 ± 0.26 vs 4.72 ± 0.27, *P* = 0.003 (6) 3.34 ± 0.31 vs 4.47 ± 0.23, *P* = 0.001
Daly et al., 2017 [23]	Acceptability questionnaires (taste, smell, texture, mouthfeel and overall acceptability)	N/A	In the GMP-AAs group, subjects took a berry flavoured GMP-AAs powder (35 g sachet = 20 g protein equivalent) which subjects prepared with water or low-protein milk	All of the subjects in the GMP-AAs group described the protein substitute as acceptable, with improved taste, mouth feel, texture, and smell compared to their conventional AAs
Pinto et al., 2017 [24]	N/A	N/A	N/A	N/A
Ahring et al., 2018 [18]	Two questions—VAS ^3^	Four DMs	DM1 = GMP; DM2 = AAs (equivalent amino acid profile as DM1); DM3 = GMP + AAs (0.16 g Phe/100 g amino acids present in GMP); DM4 = AAs (equivalent amino acid profile as DM3 but without Phe)	1) DM1: 36 ± 18, DM2: 41 ± 16, DM3: 28 ± 27, DM4: 35 ± 30); 2) DM1: 34 ± 31, DM2: 44 ± 22, DM3: 36 ± 28, DM4: 26 ± 22); all comparisons (DM1 and DM2, DM3 and DM4, DM3 to DM1 and DM2, respectively) were statistically insignificant

AAs: synthetic protein derived from L-amino acids; DM: drink mixture; GMP: glycomacropeptide; GMP-AAs: glycomacropeptide supplemented with amino acids; N/A: not available; VAS: visual analogue scale. Data are presented as mean ± standard deviation or mean ± SEs (in the case of Ney et al., 2016). ^1^ Five sensory categories—appearance, odour, taste, texture and overall acceptability (1 = dislike very much; 2 = dislike; 3 = neither like nor dislike; 4 = like; 5 = like very much). ^2^ Six questions: (1) How much do you like your AAs/GMP-AAs?; (2) How easy is it to prepare your AAs/GMP-AAs?; (3) How willing are you to take AAs/GMP-AAs three times a day?; (4) How easy is it to stay on your phenylketonuria diet when you are using AAs/GMP-AAs?; (5) How comfortable are you eating AAs/GMP-AAs in social situations?; (6) Overall, how convenient is it to take and consume AAs/GMP-AAs away from home? (1 = dislike extremely; 2 = dislike; 3 = somewhat dislike; 4 = somewhat like; 5 = like; 6 = like extremely). ^3^ Two questions: (1) How satisfied are you? and (2) How does the DM taste? This was presented to patients as a horizontal line, ranking from 0 = very hungry to 100 = very satisfied and from 0 = bad taste to 100 = good taste.

**Table 4:** Quality of all included studies according to Grading of Recommendations Assessment, Development and Evaluation (GRADE) system.

Outcomes	Number of Studies	Study Design	Risk of Bias	Inconsistency	Indirectness	Imprecision
Blood Phe	2	Randomised trials	Not serious	Serious	Not serious	Very serious
	5	Observational studies	Not serious	Serious	Not serious	Very serious
Blood Tyr	2	Randomised trials	Not serious	Serious	Not serious	Very serious
	4	Observational studies	Not serious	Serious	Not serious	Very serious
BUN	2	Randomised trials	Not serious	Serious	Not serious	Very serious
	3	Observational studies	Not serious	Serious	Not serious	Very serious
Glucose	2	Randomised trials	Not serious	Serious	Not serious	Very serious
	4	Observational studies	Not serious	Serious	Not serious	Very serious
Acceptability	2	Randomised trials	Not serious	Serious	Not serious	Very serious
	5	Observational studies	Not serious	Serious	Not serious	Very serious

BUN: blood urea nitrogen; Phe: phenylalanine; Tyr: tyrosine. The GRADE ranks as follows: not serious, serious, and very serious.

**Table 5:** Risk of bias in non-randomised studies according to the Risk of Bias in Non-Randomised Studies of Interventions (ROBINS-I) tool.

Author, year (Ref.)	Domain 1	Domain 2	Domain 3	Domain 4	Domain 5	Domain 6	Domain 7	Overall
Lim et al. 2007 * [10]	N/A	N/A	N/A	N/A	N/A	N/A	N/A	N/A
van Calcar et al. 2009 [17]	3	3	1	1	1	1	1	2—Moderate
MacLeod et al. 2010 [20]	3	3	1	1	1	1	1	2—Moderate
Zaki et al. 2016 [21]	3	3	1	1	1	3	3	2—Moderate
Daly et al. 2017 [23]	3	3	1	1	1	1	1	2—Moderate
Pinto et al. 2017 [24]	3	3	1	1	1	1	1	2—Moderate

N/A. not applicable; Domain 1: confounding; Domain 2: selection of participants; Domain 3: classification of intervention; Domain 4: deviation from interventions; Domain 5: missing outcome data; Domain 6: measurement of outcomes; Domain 7: selection of reported result; Overall. Risk of bias assessment: 0—No information; 1—Low; 2—Moderate; 3—Serious; 4—Critical. * Non-comparative study only acceptability of GMP products is evaluated, therefore this tool is not applicable in this case.
